# Prostate Cancer and Bone: The Elective Affinities

**DOI:** 10.1155/2014/167035

**Published:** 2014-05-28

**Authors:** Nadia Rucci, Adriano Angelucci

**Affiliations:** Department of Biotechnological and Applied Clinical Sciences, University of L'Aquila, 67100 L'Aquila, Italy

## Abstract

The onset of metastases dramatically changes the prognosis of prostate cancer patients, determining increased morbidity and a drastic fall in survival expectancy. Bone is a common site of metastases in few types of cancer, and it represents the most frequent metastatic site in prostate cancer. Of note, the prevalence of tumor relapse to the bone appears to be increasing over the years, likely due to a longer overall survival of prostate cancer patients. Bone tropism represents an intriguing challenge for researchers also because the preference of prostate cancer cells for the bone is the result of a sequential series of targetable molecular events. Many factors have been associated with the peculiar ability of prostate cancer cells to migrate in bone marrow and to determine mixed osteoblastic/osteolytic lesions. As anticipated by the success of current targeted therapy aimed to block bone resorption, a better understanding of molecular affinity between prostate cancer and bone microenvironment will permit us to cure bone metastasis and to improve prognosis of prostate cancer patients.

## 1. Introduction

Although only 5% of patients diagnosed with prostate cancer (PCa) have metastatic disease, more than 60% of those who die from PCa have metastases disseminated in distant sites. In early diagnosed, low grade PCa, the surgical, radiation, and hormone therapies assure a long life expectancy. However the risk for metastatic disease significantly increases as early as 2 years after surgical resection of advanced primary cancer, with more than 40% of distant recurrence [1]. In PCa patients bone represents the most common distant metastatic site and metastatic disease to the bone is the first cause of morbidity and mortality associated with PCa. Pathological fracture is associated with more than 20% increased risk of death in bone metastasizing cancers [2]. Metastatic recurrence to the bone is frequently observed along with hormonal resistance and the loss of therapeutic chances. Autopsy series have revealed that more than 90% of patients diagnosed with PCa show evidence of skeletal metastases. This is highly suggestive feature that renders PCa to be peculiar also in comparison with other osteotropic tumors, such as breast and lung cancer.

Imaging diagnosis of bone metastases in PCa patients frequently reveals extensive osteoblastic activity and histologic observation of bone biopsies confirms the increase in mineralized matrix and the presence of several mature osteoblasts adjacent to the tumor tissue in the bone [3]. Indeed, PCa is generally prone to form blastic bone lesions, with a frequency of up to 90% [4]. Osteoblastic metastases are characterized by increased abnormal bone formation, with an elevated osteoid surface. Moreover, the new bone has woven features and being of poor quality offers poor mechanical resistance. However, in PCa patients with skeletal metastases also an increase of bone resorption markers is frequently observed [5]. In fact, beside radiography, the progression of metastatic PCa is monitored by the product of collagen degradation, N-telopeptide (NTx) or C-telopeptide type I collagen (CTx), and cross-linked C-terminal telopeptides (ICTP) together with markers of bone formation, such as amino-terminal procollagen propeptides (PINP), osteocalcin, and bone-specific alkaline phosphatase (BALP) [6–8]. All these markers are strongly correlated among each other and with the prostate-specific antigen (PSA) [9]. Moreover, antiosteoclast drugs largely used in the treatment of osteolytic bone metastases are shown to be effective also against osteoblastic metastases. Markers associated with collagen degradation result in useful predictive tools in monitoring skeletal related events (SREs) in PCa patients receiving bisphosphonate therapy [10, 11]. Therefore, it seems that the prevalence of osteoblastic versus osteolytic bone metastasis is the result of the ratio between bone formation and bone degrading activities, rather than a dichotomy [12]. Currently it is believed that bone resorption is an important requisite in the progression of bone metastasis even when a net increase in new bone formations is observed. The importance of osteoclast activation in PCa associated bone metastases is evident if we consider results from current therapeutic approaches. In fact, pharmacologic osteoclast inhibition reduces significantly the median time for first SREs (pathological fracture, spinal cord compression and surgery, or radiation therapy to bone) and the number of patients who experienced an event within 2 years [13]. In addition, preclinical models using PCa cell lines are associated with a prevalent osteolytic phenotype and several in vitro evidences suggest that PCa cells are able to modulate directly osteoclast activity. Based on these data, it is clear that a better understanding of the determinants underlying molecular affinities between PCa cell phenotype and bone physiology is needed.

## 2. Osteoclasts, Osteoblasts, and Bone Remodeling

Contrary to what appears, bone is a dynamic tissue, having as its unique feature the ability to destroy and rebuild itself during the lifetime of each individual by a physiological process named bone remodeling [14]. The reasons for this self-injurious behavior, which is also energy consuming, are of course well known and justified, since bone remodeling allows the regulation of calcium homeostasis, the repair of microfractured or ischemic bone, and the substitution of infantile woven bone with a mechanically competent bone.

Bone remodeling relies on two principal cells of the bone tissue, osteoblasts and osteoclasts, whose functions are subjected to a fine tune regulation in order to preserve a correct bone mass. Another player involved in this process is the osteocyte, a cell arising from the osteoblast after its entrapment in the bone matrix at the end of the bone formation phase. Osteocytes also participate in this bone remodeling process, with roles of mechanosensing and of coordination of osteoblast and osteoclast functions.

### 2.1. Osteoblast Differentiation

Osteoblasts are cells of mesenchymal origin, arising from pluripotent mesenchymal stem cells (MSCs) that, according to the selective expression of specific genes, may give rise to different tissue specific cells including osteoblasts, chondrocytes, fibroblasts, myocytes, and adipocytes [15].

The first step of differentiation is therefore the commitment of MSCs towards an osteo/chondroprogenitor, which is characterized by the expression of two pivotal transcription factors: runt-related transcription factor 2 (runx2) and osterix, the latter being activated according to a runx2-dependent or independent signaling. To be committed, osteoblasts relies on two main pathways which are activated since the early steps of their differentiation: wingless-type protein (Wnt) and bone morphogenetic proteins (BMPs) pathways, whose deregulation has been called into question in the development of PCa bone metastases, as will be discussed later.

Runx2 expression further promotes the commitment of the osteo/chondroprogenitor towards an osteoblast phenotype, giving rise to a pool of osteoprogenitors that acquire alkaline phosphatase (ALP) expression and undergo a proliferative stage. We have now a preosteoblast, which undergoes morphological changes, thus lacking a spindle-shaped feature and becoming a large cuboidal osteoblast, with high levels of ALP activity and secreting the bone matrix proteins bone sialoprotein (BSP) II, osteopontin (OPN), fibronectin, and collagen I. The late stage of differentiation is characterized by the highest levels of osteocalcin (OC), which is a marker of mature osteoblasts and by the switch on of specific genes involved in bone mineralization (for review see [14]).

### 2.2. Osteoclast Differentiation

Osteoclasts, the multinucleated cells devoted to resorb bone, arise from the monocyte/macrophage hematopoietic lineage [16]. One of the earliest molecules activated to promote osteoclast commitment is the transcription factor PU.1, whose expression increases as marrow macrophages differentiate into osteoclasts [17]. PU.1 in turn positively regulates the expression of the receptor of macrophage-colony stimulating factor (M-CSF), named c-fms, as well as of the receptor activator of nuclear factor Kappa B (RANK) that, upon binding to its ligand RANKL, triggers the fusion and differentiation of osteoclast precursors into mature osteoclasts [18]. Other transcription factors involved in osteoclast differentiation are microphthalmia-associated transcription factor (MITF) and nuclear factor of activated T-cell, cytoplasmic 1 (NFATc1), both forming a complex with PU.1, thus promoting the transcriptional expression of osteoclastogenic genes [19, 20].

## 3. The Bone Remodeling Unit

Bone remodeling is a multicellular and multispatial event that takes place with the close cooperation among the cells that constitute the so-called basic multicellular unit (BMU), which includes osteoclasts, osteoblasts, osteocytes embedded in the bone matrix, the bone lining cells, and the capillary blood supply (reviewed in [21]). The following steps achieve the bone remodeling virtuous cycle.

### 3.1. Activation Phase

Before this phase, bone surface is in a “resting condition,” being covered by a layer of quiescent osteoblasts called lining cells. Different stimuli, such as changes in mechanical loading perceived by the osteocytes, specific paracrine/systemic factors released in the bone microenvironment, or the occurrence of a microfracture all activate the lining cells, which increase their own surface expression of RANKL, thus promoting preosteoclasts differentiation toward mature and active osteoclasts.

### 3.2. Resorption Phase

Once differentiated, osteoclasts closely adhere to the bone surface and resorb it, first by releasing protons that acidify the milieu, thus allowing solubilization of the inorganic part (hydroxyapatite), and then by releasing enzymes (i.e., MMP-9 and cathepsin K), that degrade the organic components of the matrix. This phase is quite fast and must be appropriately switched off, to avoid an exacerbated bone resorption. Therefore, once they accomplished their function, osteoclasts undergo apoptosis, thus leaving the place to the next phase.

### 3.3. Reversal Phase

This phase is characterized by the involvement of the reverse cells, whose role has not been completely explained. Indeed, it is known that they are macrophage-like cells with a likely function of removal of debris produced during bone matrix degradation. Therefore, this phase is of transition from osteoclast to osteoblast activity.

### 3.4. Formation Phase

This phase is triggered by the previous release of the factors usually stored in the bone matrix such as transforming growth factor beta (TGF*β*), Insulin-like growth factors (IGF) I and II, fibroblast growth factors (FGFs), platelet-derived growth factor (PDGF), and BMPs. These factors retrieve the osteoblasts in the reabsorbed area, eventually leading to the deposition of new bone matrix, initially not calcified (osteoid). Osteoblasts also promote bone matrix mineralization, thus closing the virtuous circle of bone remodeling.

Several factors, systemic and paracrine, regulate bone remodeling; this regulation being also achieved by a mutual cross-talk between osteoclasts and osteoblasts. Indeed, osteoblasts and osteocytes produce the proosteoclastogenic cytokine RANKL that, as above mentioned, binds to its receptor RANK expressed by osteoclast precursors, thus activating intracellular signaling leading to osteoclast differentiation [22]. Osteoblasts also secrete OPG, which by binding to RANKL inhibits osteoclast differentiation, because it acts as a decoy receptor having the same structure of the extracellular portion of RANK. Therefore, a balanced osteoclast differentiation relies on a correct RANKL/OPG ratio. Other paracrine factors produced by osteoblasts that stimulate osteoclast formation are interleukin-6 (IL-6), IL-1*β*, and tumor necrosis factor-*α* (TNF-*α*).

As will be described in more detail, tumor cells growing in the bone marrow induce a drastic deregulation of bone remodeling, by producing several paracrine factors that in turn influence osteoclast and osteoblast functions.

## 4. Theories Underlying PCa Osteotropism

**Figure 1:**
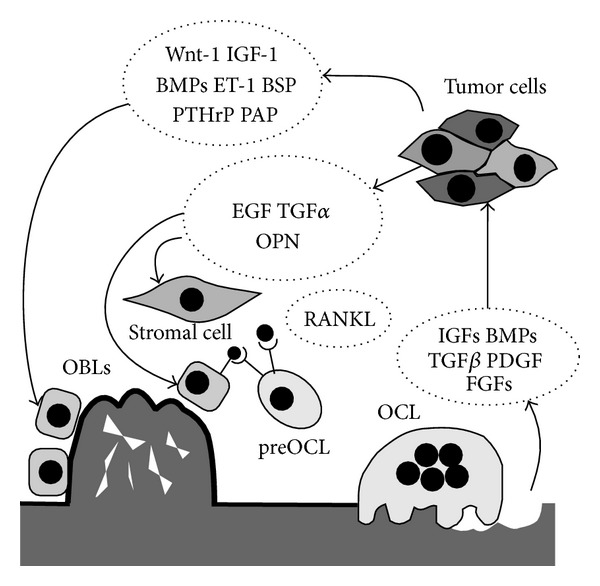
Schematic representation of molecular pathogenesis of osteoblastic metastases in prostatecancer. Once reached the bone, tumor cells produce factors that stimulate osteoblasts (OBLs) differentiation and activity. Tumor cells also produce factors that act on stromal cells and osteoblasts that in turn stimulate osteoclasts (OCL) differentiation, thus perturbing bone remodeling on both sides. Finally, osteoclasts resorb bone allowing the release of growth factors that stimulate tumor cells survival and growth, thus fuelling the vicious circle.

The molecular features that render bone as a preferred site for PCa growth should be evaluated considering the current knowledge of the metastatic process. From a biological point of view, the metastasis is a very inefficient process, since it has been estimated that less than 0.1% of tumor cells, once detached from the primary site, are able to reach and colonize a distant organ [23]. Several steps in cancer dissemination are restrictive and although the invasive capacity acquired in the primary site by cancer cells permits their extravasation, not all of them are adapted to survive in circulation, to escape from the bloodstream and to seed secondary growth in distant organs. In many cases metastatic distribution could be explained by hematologic considerations, so that highly perfused organs with dense vasculature favor tumor cell embolization, arrest in that site, and secondary growth. Indeed, lung is the first vasculature bed encountered by tumor cells after venous spreading and this is in agreement with the fact that lung is one of the most common sites of metastasis. In PCa the high incidence of spinal cord metastases could be partially caused by cancer cell dissemination through Batson's plexus of veins: venous blood from pelvis flows not only into the venae cavae but also into a vertebral-venous plexus that extends from the pelvis throughout the epidural and perivertebral veins. However, other organs localized in pelvis that utilize this venous circulation, including colon-rectum, fail to demonstrate a comparable affinity for bone. Thus the high osteotropic potential of PCa cells is better explained by the metastatic homing theory proposed in the first version by Paget and revisited in molecular terms by extensive experimental evidence since early 1980s [24]. This theory was aimed at clarifing the high predilection for a metastatic site that resulted higher than expected by hematologic considerations. The multistep hypothesis postulates that organ preference could be established in one of the several phases of cancer cells dissemination, from intravasation to terminal growth in distant site, and it strongly supports the current use of targeted therapy in metastatic settings [25]. The bone could be a “fertile” soil because it is a reservoir of growth factors; however, these factors are not immediately available because they are stored in the mineralized matrix or present in inactive form. In addition, bone is also a peculiar “soil” for cancer cells that is characterized by one important adverse feature: the space available for cell growth is delimited by rigid boundaries that cannot be digested directly by cancer cells. In order to stimulate bone remodeling and free growth factors, cancer cells secrete many factors that act on osteoclasts and osteoblasts. This model was called “vicious cycle” and postulates the presence of a self-sustaining reciprocal stimulation between cancer and bone cells [26] (Figure 1). More recently, as a natural extension of vicious cycle it was suggested that PCa cells develop a bone-restricted phenotype. This theory, called osteomimicry, was proposed by Koeneman et al. and was based upon the hypothesis that prostate cells must acquire “bone-like” properties in order to adapt and grow in the bone environment [27]. In particular, the PCa cells express proteins associated with osteoblast maturation and differentiation, including BMPs, parathyroid hormone related protein (PTHrP) and endothelin-1 (ET-1) (Figure 1). The factors released by PCa cells do not generally confer a growth advantage to tumor cells without stimulating also bone cell functions (for review, [28]). Other exemplificative osteoblast-related factors expressed by tumor cells are Runx2 and ALP. To date the molecular bases of PCa osteotropism have not been completely elucidated but important cues are derived from studies in animal models.

## 5. Issues from Preclinical Studies

The demonstration of bone homing in PCa preclinical studies has encountered several difficulties due to limitations in the available cell lines and to technical problems in reproducing bone metastases in vivo. Genetically engineered models (GEMs) of PCa were established by either the overexpression of an oncogene controlled by a prostate-specific promoter or the targeted deletion of specific genes. The best characterized GEMs have been created using the androgen-dependent probasin promoter to determine tissue specific transcription of SV40 oncogenes: the transgenic adenocarcinoma of the mouse prostate (TRAMP) model in which the transgene includes large and small T antigen early SV40 oncogenes [29] and the LADY and the TRAP rat models, which express only the large T antigen [30, 31]. These animal models recapitulate only partially the progression of the human disease, and although GEM models of mouse or rat PCa commonly demonstrate metastasis to lymph node and lung, bone metastasis in these models is an occasional event [32, 33]. Available human PCa cell lines are derived from advanced diseases and they are hormone refractory and highly tumorigenic in immunocompromised mice. However, when these cells are injected intravenously (i.v.) in the tail vein of severe combined immuno deficiency (SCID) mice, cancer cells are initially able to metastasize to a wide variety of tissues [34]. Therefore, tail vein injection does not produce a preferential tropism to bone [35]. In order to divert cells into the vertebral venous plexus, injection into the tail vein was performed, while the inferior vena cava was occluded [36]. In this model the incidence of lung metastases was significantly decreased as compared with nonoccluded control mice and bone metastases developed in about 50% of animals.

Difficulties in realizing an adequate in vivo model could depend on the following:

- the preferential entrapment of cancer cells in the capillary bed of highly perfused organs;
- the long latency time in natural development of bone metastases;
- the phenotypic modification induced by in vitro culture of cancer cell lines;
- species-specific features.

The hypothesis of human-specificity of metastatic models was suggested by Nemeth et al., who observed that the i.v. injected human PCa cells preferentially “homed” to subcutaneous transplanted human fetal bone with respect to transplanted murine fetal bone [37]. At present, effective bone metastatic models are finalized to bypass the pulmonary vasculature, including direct injection into medullary cavity of bones [38], and injection into the left cardiac ventricle [39]. These techniques dramatically increase the incidence of bone metastases and by their utilization, it has been demonstrated that human PCa cell lines can grow in bone medulla and interact with mouse bone cells, reproducing the pathogenesis observed in PCa metastatic patients. Indeed, in a very interesting way, increased osseous metastasis frequency by PCa cell lines has been achieved by serial in vivo selections. Several studies have demonstrated that serial selections of PC3 sublines established from explant cultures of bone lesions promote osteotropic potential of the initial cell population [36, 40, 41]. Thalmann et al., utilizing this model, were able to select a LNCaP subline with acquired metastatic potential to bone, although the incidence of bone metastases was less than 50% [42]. Also when injected directly in bone marrow cavity these LNCaP sublines showed a minor tumor “take” rate with respect to PC3 cells. Importantly, LNCaP subpopulations were able to produce osteoblastic lesions [43]. More recently, the human PCa xenograft line, LuCaP 23.1, has been described as osteoblastic when injected directly into tibia of SCID mice [44]. The experimental procedure of serial selections is potentially a very fertile field of investigation for molecular determinants of bone tropism. Highly osteotropic LNCaP derivatives present several features that differ from parental low metastatic LNCaP cells, including progression toward androgen-independent state, expression of bone matrix proteins, and increased invasive potential [45]. The characterization of these models has permitted to identify several proteins associated with metastatic PCa growth and bone remodeling, including osteoprotegerin (OPG), RANK ligand, PTHrP, and ET-1 [46]. The prevalence of osteosclerosis over osteolysis appears dependent on the ability of PCa cells to release paracrine factors that in turn can drive osteoblast differentiation and function. In particular, Wnt-1, IGF-1, BMPs, ET-1, and PTHrP are some of the factors called into question in the development of osteoblastic metastases [47]. Consistently, a recent work from Larson et al. showed that PCa-derived prostatic acid phosphatase (PAP), a protein secreted by normal prostate and PCa cells, is highly expressed in osteoblastic bone metastases and promotes mineralization in human and mouse derived osteoblasts [46]. A mutual crosstalk between osteoblasts and PCa cells has been recently described [48]. Indeed, Hagberg Thulin and colleagues not only demonstrated that the castration-resistant prostate cancer cell line LNCaP-19 expressed a pronounced osteogenic phenotype compared to the androgen-dependent cell line LNCaP, but also that its ability to reproduce osteosclerotic metastases in intratibial injected mice is promoted by osteoblast-derived factors. In addition, C4-2B cells, a bone-homing cell subpopulation of LNCaP cells, were found to induce mineralization in vitro under appropriate conditions [49].

**Figure 2:**
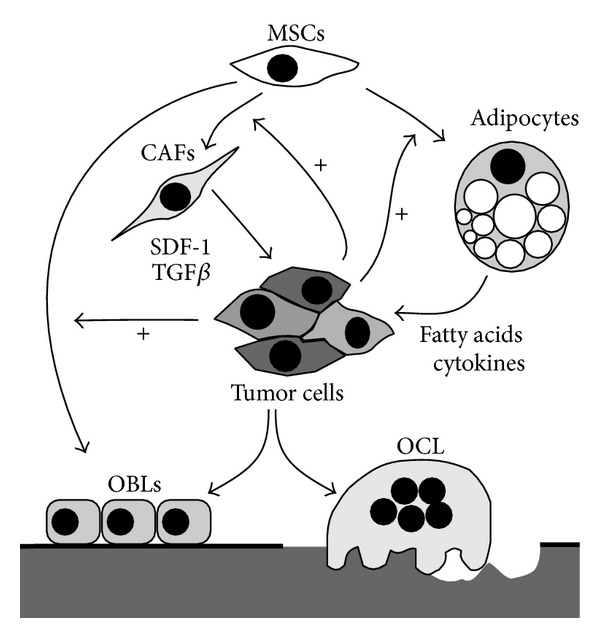
Proposed metastatic stroma associated with prostate cancer growth in the bone. Cancer cells can stimulate mesenchymal stem cells (MSCs) to differentiate in osteoblasts, fibroblasts, and adipocytes. Cancer associated fibroblasts (CAFs) release factors, including SDF-1 and TGF*β*, which stimulate cancer cell invasion and proliferation. In addition, cancer associated adipocytes, through the release of lipids and cytokines, can sustain cancer growth and induce an osteotropic phenotype. These interactions could influence the crosstalk between cancer cells and osteoblasts/osteoclasts (OBLs, OCL) and the formation of bone lesions. The increased differentiation of MSCs in osteoblasts induced by PCa cells could shift the balance of bone homeostasis towards aberrant mineralization.

Most of the researchers have modeled bone metastasis in vitro by examining interactions between PCa cells and bone cells. The bulk of data has been produced with the osteoclasts and the osteoblasts for their unique role in regulating the remodeling of the mineralized tissue. However bone marrow is a very heterogeneous tissue and other differentiated cell types, including fibroblasts and adipocytes, can contribute to the formation of PCa stroma in bone metastasis [50] (Figure 2). In addition, bone marrow is the house of two major types of stem cells: the hematopoietic stem cells (HSCs) and mesenchymal stem cells (MSCs). In the last years, MSCs have attracted attention for their capacity, under proper culture conditions, of differentiating into a variety of mesodermal lineages, including fibroblasts, chondrocytes, osteoblasts, and adipocytes. Indeed, while the multilineage differentiative ability of MSCs is relatively easy to demonstrate in vitro, it is difficult to reproduce and/or determine in vivo. This aspect could explain the conflicting results regarding the influence of MSCs in different models of PCa progression [51]. An intriguing but debated aspect is about the possibility that PCa cells can induce MSCs to differentiate in osteoblasts. Available data suggest that conditioned medium from metastatic PCa cell line favors commitment of MSCs toward osteoblasts, while a nonmetastatic PCa cell line fails to induce osteoblast differentiation in the same culture conditions [52]. In agreement with this observation, it has been shown that intratibial injected MSCs stimulate new bone formation only when coinjected with the osteotropic cell line PC3. However in the same model MSCs injected into already established bone metastasis suppressed PCa growth [51]. MSCs differentiation may also contribute to form cancer associated fibroblasts (CAFs) in bone metastases [53]. Although the presence of myofibroblasts and fibroblasts in reactive stroma of primary PCa is fundamental in cancer progression, little is reported on CAFs in PCa bone metastases. The cell origin of prostate CAFs in bone metastases is not precisely known, but the suggestive hypothesis of primary prostate CAFs metastasizing with PCa cells to the bone is currently not supported by evidence. It was recently proposed that MSCs are induced to form CAFs, which secrete stromal, cell derived factor-1 (SDF-1/CXCL12) (Figure 2). SDF-1 in turn binds to CXCR4 on tumor cell surface and induces epithelial-to-mesenchymal transition [54]. The similarity of reactive stroma in primary and bone metastatic site could drive the coevolution of PCa toward an osteotropic phenotype stimulating the expression of the cytokines CXCL1 and CXCL16 [55]. In bone tissue associated with PCa, CAFs express the myofibroblast marker *α*-SMA, and the androgen receptor (AR). As observed in primary cancer, TGF*β* receptor II (TGF*β*RII) expression was lost in CAFs in the majority of bone metastatic tissues examined [55]. The loss of TGF*β* sensitivity in prostatic CAFs promoted PCa cell proliferation and invasion in a xenograft model [56]. Bone CAFs have increased levels of chemokine (C-C motif) ligand (CCL) 5, chemokine (C-X-C motif) ligand (CXCL)5, versican, tenascin, connective tissue growth factor (CTGF), SDF-1, and hypoxia inducible factor 1-*α* (HIF-1*α*) [57].

Another intriguing but largely unexplored hypothesis suggests that MSCs could sustain PCa growth by differentiating in adipocyte lineage. In agreement with this hypothesis, PCa cells localize to lipid-rich regions in bone marrow metastases and interact with adipocytes in vitro with a resultant increase in their proliferation [58]. Adipocyte numbers in the bone marrow strongly correlate with age; thus, bone metastatic disease develops in adipocyte-enriched environment [59]. The homing to the bone could be initially favored by a direct attraction by lipids released from bone marrow adipocytes [60]. In addition, within bone marrow, interaction between the two cell types results in translocation of adipocyte-stored lipids to the metastatic tumor cells [61]. Specific lipids have been shown to be key factors for PCa cells progression, working as pleiotropic factors able to stimulate proliferation, gene expression, chemotaxis, and energy metabolism. Arachidonic acid (AA) and its precursor linoleic acid can directly stimulate in vitro growth of human PCa cells. Cancer cells could utilize adipocytes in bone marrow as lipids storehouse (Figure 2). Consistently, deprivation of adipocytes in the bone marrow reduces the homing of PCa cells to bone marrow in an AA-dependent manner [58, 60]. An AA-rich environment could sustain the perturbation of bone remodeling. In fact, it was demonstrated that metabolites of AA stimulate the release by cancer cells of cytokines that in turn favor osteoblast differentiation [62] (Figure 2). In addition to fatty acids, other factors released by bone marrow adipocytes can influence PCa phenotype, including proinflammatory cytokines and adipokines; however, the available data are at present still inconsistent.

## 6. Current Therapeutic Options

The agents currently used for treating bone metastatic PCa can be divided in four categories: chemotherapeutic drugs (docetaxel [63], cabazitaxel [22]); agents targeting bone homeostasis (bisphosphonates, denosumab); antiandrogen drugs; and radiopharmaceuticals (radium-223 [64]). Over the past two decades, the bisphosphonates have emerged as safe and effective components of treatment in bone metastatic disease from different cancers. Their use in managing bone metastases has had a profound beneficial effect on the frequency and severity of skeletal morbidity, eventually leading to an improvement of the quality of life [11]. Bisphosphonates bind to exposed bone mineral and then are internalized by the osteoclasts, thus affecting their homeostasis and bone resorption activity. The nitrogen-containing bisphosphonates (N-BPs, eg., zoledronic acid, pamidronate) act by interfering with the farnesyl pyrophosphate synthase (FPPS), a key enzyme in the mevalonate pathway, eventually leading to the block of the covalent attachment of isoprenyl chains to small guanosine triphosphatases, which in turn inhibits their intracellular localization and functions in osteoclasts. Moreover, the disruption of the mevalonate pathway by N-BPs determines the accumulation of a cytotoxic adenosine triphosphate analog called ApppI (triphosphoric acid I-adenosin-5′-yl ester 3-(3-methylbut-3-enyl) ester) which in turn induces apoptosis [65, 66]. Zoledronic acid is effective in preventing and delaying SREs in several solid tumors, including castrate-resistant PCa (CRPC) [67]. Preclinical and initial clinical evidence has suggested that zoledronic acid could offer benefits also in improving SREs in men with high risk for metastatic disease; however, the efficacy of zoledronic acid in this clinical situation is currently debated [68–70].

Denosumab (AMG 162) is a humanised monoclonal antibody targeting RANK/RANKL pathway. United States and European regulatory agencies approved the use of denosumab for both metastatic castration-sensitive and resistant PCa patients. Denosumab has long half-life and achieves rapid and prolonged bone resorption inhibition after injection in a single subcutaneous dose. It acts by binding to RANKL, thus avoiding its interaction with RANK, eventually leading to the inhibition of formation, activation, and survival of osteoclasts [71]. Denosumab has been shown to delay the onset of bone metastases more consistently with respect to bisphosphonates [72]. In fact, it was observed that the NTx marker normalized more frequently with denosumab than with bisphosphonate therapy [73]. However, both zoledronic acid and denosumab offer a few months advantage over placebo to prolong time to first SRE [74]. Moreover, side effects have been detected for both compounds, such as osteonecrosis of the jaw and hypocalcemia, while zoledronic acid has been associated with deterioration of renal function, and therefore it cannot be used in presence of severe renal insufficiency [75].

For PCa patients hormone ablation is the common therapeutic option. However, resistance to this therapy frequently occurs and metastatic disease is associated with the progression towards a castrate-resistant stage (CRCP). Until few years ago, no androgen-deprivation agents have been convincingly shown to improve outcomes among metastatic CRCP patients. Notably, androgen deprivation therapy itself is a potential cause of loss of bone mineral density and can favor an increased incidence of fractures. However several recent studies have found the AR to remain a potential therapeutic target throughout the progression of PCa and the recent phase III clinical trials have demonstrated that novel potent androgen-directed agents carry benefit in reducing SREs over that of bisphosphonates alone [76]. Among them the enzalutamide, which has demonstrated a significant improved median overall survival in men with CRPC [77], as well as an increase in the radiographic progression-free survival in comparison with the placebo arm (8.3 versus 2.9 months) [78]. Abiraterone acetate, a selective oral inhibitor of cytochrome P450 (CYP17), a key enzyme in the production of androgens, estrogens, and glucocorticoids, has been initially approved for the treatment of patients with CRPC who received prior docetaxel chemotherapy. Recent results from large phase III clinical trial indicated that abiraterone acetate in chemonaïve patients determined a median radiographic progression-free survival of 16.5 months with respect to 8.3 months in the arm treated with prednisone alone and improved palliation of pain [79]. Based on these data, abiraterone acetate has been approved for treatment also in the prechemotherapy phase of CRPC. The new hormone ablation options denote an important step forward for the control of the disease. However, in order to maximize the prevention of bone lesions, avoiding the possible phenomena of new resistance, the use of adjuvant bone remodeling-targeted therapy is desirable.

## 7. Targetable Signaling Pathways Involved in PCa Osteotropism

### 7.1. Wnt Signaling

Wnt family accounts for at least 19 glycoproteins characterized by a conserved pattern of 23/24 cysteine residues and they are classified into canonical and noncanonical signaling molecules according to the downstream pathway that is activated [80]. Although Wnt pathway is mainly involved in organ development and tissue homeostasis, several evidences show that it also has a role in tumorigenesis [81, 82]. Wnt canonical pathway (also known as Wnt/*β*-catenin pathway) is triggered by binding of Wnt protein to frizzled and LRP5/6 transmembrane receptor complex, which in turn inactivates the enzyme glycogen synthase kinase 3*β* (GSK3*β*) by interacting with Axin, Frat-1, and disheveled (Dvl) proteins, thus preventing *β*-catenin phosphorylation. In its hypophosphorylated form, *β*-catenin is more stable, thus accumulating to the cytoplasm and translocating to the nucleus. Here it interacts with the Tcf/Lef family of transcription factors and regulates transcriptional expression of Wnt target genes. In contrast, GSK3*β* activity induces *β*-catenin phosphorylation, thus targeting the protein to proteasome ubiquitination [83, 84]. The interest for Wnt canonical signaling in the study of bone physiology came from the evidence that gain of function mutations in LRP5 receptor led to high bone mass (HBM) syndrome [85], while disrupted Wnt signaling was linked to bone loss diseases, including osteoporosis, van Buchem disease, and sclerosteosis [86, 87]. Indeed, it has been shown that Wnt signaling plays a pivotal role for differentiation of osteoblasts from progenitor cells. Two families of inhibitors regulated Wnt pathway: dickkopf (DKKs) and secreted frizzled-related proteins (sFRP). DKKs interact with LRP5/6 and kremen; then this complex is internalized, ubiquitinated, and degraded by the proteasome. Similar to DKK1 is sclerostin, alias Wnt inhibitor factor 1, which is predominantly produced by osteocytes. Also sclerostin interacts with LRP5/6 and kremen thus promoting the degradation of this complex [88]. As far as sFRP are concerned, they inhibited Wnt pathway by sequestering Wnt from binding to FRZ receptors or by directly binding to FRZ [88]. Given the pivotal role of Wnt in osteoblast differentiation, this pathway has been investigated as one of the candidates involved in osteoblastic bone metastases induced by PCa cells. Indeed, PCa cells express several molecules belonging to Wnt superfamily, such as Wnt3a, Wnt7b, and Wnt10b, which are strongly involved in the early phases of osteoblast differentiation. In contrast, PCa cell lines that in animal models usually develop osteolytic bone metastases were found to express increased levels of the Wnt inhibitor DKK-1, compared to less aggressive PCa cells that produced mixed lesions. Moreover, it has been found that DKK-1 expression was significantly increased in primary PCa compared with nonneoplastic tissue, while it was reduced in PCa-induced bone metastases compared to primary tumors [89]. Conversely, inhibition of DKK-1 expression by siRNA in osteolytic PC3 cells promoted osteoblast mineralization in coculture experiments [90]. Interestingly it has been demonstrated that MSCs involvement in bone metastasis could be dependent upon Wnt signaling. In fact Wnt7b is a mediator of MSC differentiation and it was proposed as an important mechanism in the development of the osteoblastic bone response [91]. However, because MSCs are potential source of DKK-1, more investigation is required to define how MSCs contribute to Wnt signaling in the bone metastasis [92].

Taken together, all these data indicate that a deregulation of Wnt pathway plays a crucial role in PCa bone metastases and that an unbalance between factors promoting and inhibiting Wnt signaling could determine the type of the prevalent bone lesion induced by PCa cells [89].

### 7.2. ErbB Receptors

**Figure 3:**
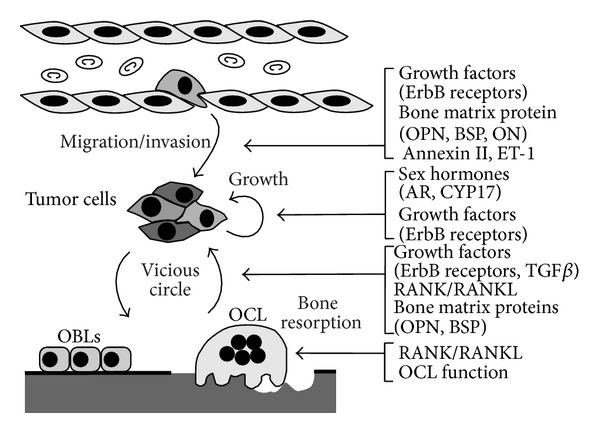
Potential therapeutic targets in the progression of bone metastasis in prostate cancer. In the figure the main final metastatic steps are schematically illustrated, including extravasation and secondary growth (see details in the text).

Several in vitro studies have indicated that activation of ErbB receptors is a potent stimulus for survival, proliferation, migration, and invasion of PCa cells. In addition, numerous preclinical studies have proposed a leading role for ErbB kinases in the progression of bone metastases [93] (Figure 3). The family of ErbB receptors includes four members: ErbB1 (epidermal growth factor receptor, EGFR); ErbB2 (neuroblastoma or glioblastoma-derived, NEU; human epidermal growth factor receptor 2, HER2); ErbB3 (human epidermal growth factor receptor 3, HER3); ErbB4 (human epidermal growth factor receptor 4, HER4); however, only for EGFR and HER2 sufficient data have been accumulated for suggesting a role in bone metastasis. HER2 is an effective therapeutic target in breast cancer, and although HER2 expression does not appear to be associated with osteotropism, clinical trials have demonstrated the efficacy of HER2 inhibitors in delaying skeletal events [94]. HER2 receptor, which is frequently overexpressed in breast cancer, is a preferential partner of EGFR and the formation of EGFR-HER2 heterodimers potentiates EGFR function by increasing EGF binding affinity, stabilizing and recycling the receptor on the membrane, and expanding the repertoire of intracellular signaling responses [95]. However, EGFR and HER2 are rarely found overexpressed or mutate in PCa, suggesting that their role in bone osteotropism is realized through collaboration with other oncogenic determinants. The chemical inhibition of EGFR is able to suppress the dissemination of PCa cell line in an animal model of bone metastasis [96]. The inhibition was more evident in highly osteotropic PCa sublines and correlated with increased expression of the urokinase plasminogen activator receptor (uPAR). The phenomenon of molecular collaboration of EGFR with other partners on cell membrane represents an important feature of cancer progression and may in part explain why targeted therapy designed to circumvent EGFR signaling has yielded only modest clinical success in cancer patients [93]. Interestingly, the bone matrix protein OPN has been suggested to stimulate PCa cell proliferation by enhancing the association of its receptor integrin *β*1 with the EGFR, thus stimulating the sustained phosphorylation/activation of EGFR [97] (Figure 3).

EGFR may also contribute to fuel the vicious cycle. In fact, EGFR ligands released from the bone matrix after its resorption or secreted by tumor cells influence the proliferation and activity of bone cells (Figure 3). Among the recognized ligands of the ErbB family, epidermal growth factor (EGF), TGF*α*, and amphiregulin (AREG) have been proposed as main players in bone environment. EGF and TGF*α*-enriched bone microenvironment are associated with stimulation of osteoclastogenesis through increased production of RANKL by bone stromal cells [98, 99]. In addition, EGFR is expressed by osteoblasts, and upon activation it stimulates osteoblast proliferation and decreases mineralization [100].

A role of ErbB ligands in the paracrine crosstalk between PCa cells and MSCs has been postulated by recent studies. It is well known that MSCs produce a variety of growth factors and cytokines that are normally involved in inflammation and wound healing. Conditioned medium from PC3 stimulated CCL5 secretion by MSCs, and the treatment of MSCs with exogenous TGF*α* produced a significant increase in the release of other angiogenic growth factors, such as angiopoietin-2, granulocyte-colony stimulating factor, hepatocyte growth factor, IL-6, IL-8, and platelet-derived growth factor-BB [101, 102]. The use of EGFR inhibitors was effective in reducing this interaction, and it was proposed as a novel application for this class of targeted drugs [101].

### 7.3. BMPs/TGF*β*

TGF*β* has been involved in different steps of tumorigenesis and metastasis, with functions somewhere controversial. TGF*β* is a member of the TGF*β* superfamily along with BMPs, the latter having a crucial role in bone formation. Indeed, several studies showed expression of BMP-2/4,-6 and 7 in PCa bone metastases. It is well known that BMP-6 is highly expressed in PCa that have metastasized, but not in the organ confined disease, and its expression in primary prostate tumors correlates with increased recurrence rates and decreased survival [27]. More recently, it has been shown that PCa cell lines that develop osteosclerotic metastases mainly express BMP-6 [103]. Consistently, the expression of the BMP antagonist noggin seems to be restricted to those tumor cells that preferentially induce osteolytic bone metastasis, while the forced expression of noggin in osteosclerotic PCa cells inhibited their ability to induce osteoblast metastases [104].

With regards to TGF*β*, in physiologic conditions it acts prevalently as modulator of bone deposition, by stimulating migration, proliferation, and survival of osteoblasts, while its tumorigenic properties promote PCa cell migration and metastasis [105]. Moreover, TGF*β* released in the bone microenvironment appears to stimulate bone metastases by inducing proosteolytic gene expression in osteotropic cancer cells. TGF*β* increases PTHrP production, TGFb increases PTHrP production, and for this reason plasma PTHrP concentration is frequently increased in metastatic PCa patients [106] (Figures 1 and 2). Other factors regulated by TGF*β* in PCa cells and whose expression is higher in bone metastases than in primary site are cyclooxygenase-2 (COX-2) [107] and VEGF [108].

### 7.4. Endothelin-1

It belongs to the endothelins (ET) family, a group of proteins that act through G-protein coupled receptors. The involvement of ET-1 in PCa bone metastases is quite recent because, as suggested by the name, ET-1 has been initially identified as a potent vasoconstrictor and deregulation of the ET pathway contributes to cardiovascular disease. A general role for ET-1 in the development of several cancers has also been widely reported, through the promotion of tumor cell migration, invasion, proliferation, epithelial-mesenchymal transition, and angiogenesis [109] (Figure 3). In vitro, ET-1 is able to stimulate proliferation of human PCa cell lines. In addition, advanced PCa cases are frequently associated with elevated levels of plasma ET-1 and increased cancer tissue expression [48, 110]. Interestingly, ET-1 has also been associated with PCa growth in bone, where it stimulates osteoblasts proliferation and function, and reduces osteoclasts activity [111–113] (Figure 1). Moreover, ET-1, together with TGF*β*1, reduced DKK-1 expression in human stromal cells and osteoblasts, thus indirectly promoting their differentiation [114, 115]. All these findings led to the development of specific inhibitors of ET-1 signaling, which should have the double benefit of targeting PCa cells and their ability to colonize the bone.

### 7.5. Bone Extracellular Matrix Proteins

Osteomimicry hypothesis is strongly sustained by the in vitro and in vivo evidence that during progression PCa cells express several noncollagenous bone matrix proteins, including small integrin-binding ligand N-linked glycoproteins (SIBLINGs) [116, 117], OC [118], and Osteonectin (ON) [27].

SIBLINGs comprise five functionally heterogeneous secreted glycophosphoproteins, that is, OPN, BSP, dentin matrix protein 1 (DMP1), dentin sialophosphoprotein (DSPP), and matrix extracellular phosphoglycoprotein (MEPE), which are highly expressed in bone where they function as signal transducers to promote cell adhesion, motility, and survival [119]. OPN and BSP are the only SIBLING proteins for which there are sufficient evidence for a role in cancer progression and bone metastases (Figure 3). Both BSP and OPN have been proposed as specific predictive serum markers for osteotropic prostate, breast, and lung cancer, as well as for nonosteotropic cancers [120]. Indeed, OPN has a role in many different physiological settings, including immune response, wound repair, vascular homeostasis, bone remodeling, and renal functions [121]. Increasing levels of OPN expression are not limited to metastatic phase but have also been found in multistage carcinogenesis [122]. In mineralized tissues, OPN is secreted by both osteoblasts and osteoclasts in electron-dense layer of organic material situated between the mineralized and nonmineralized parts of the bones, lamina limitans, and particularly in the sites of new bone deposition [123]. As cytokine, OPN promotes resorption, inhibiting mineral deposition [124], stimulating osteoclastogenesis, and facilitating osteoclast migration and adhesion. However, osteoclastogenic activity of OPN is seen only in particular conditions, including postmenopausal osteoporosis and lack of mechanical stress [125, 126]. Indirect evidence, through knockdown of Runx2 expression, suggests that also in osteotropic malignancies the increased expression of OPN by cancer cells is permissive in determining the osteolytic disease [127]. OPN is expressed and released by PCa cells and its expression was positively correlated with PCa osteotropism [117]. Metastatic role of OPN could be accomplished through a direct action on PCa cells, in an autocrine manner, conferring a better adhesive and invasive performance to cancer cells [97, 128] (Figure 3). Most of these activities are known to be stimulated by binding to a number of different integrins via the RGD sequence (*α*v*β*3, *α*v*β*1, *α*v*β*5) or via RGD-independent interactions (*α*4*β*1, *α*9*β*1, and *α*8*β*1). OPN can also interact with specific splice variants of CD44 that are expressed by cancer cells and this interaction seems to enhance integrin activation through a mechanism of inside-out signaling [129].

BSP is produced by osteoblasts, osteoclasts, and osteocytes during bone morphogenesis and homeostasis. The prevalent activity of BSP is to facilitate bone mineralization and to stimulate osteoblast differentiation and bone repair [130]. However BSP can also induce NFkB-dependent bone resorption by stimulating osteoclast survival and osteoclastogenesis [131]. BSP is aberrantly expressed in a variety of osteotropic tumors and in breast and PCa cells elevated BSP expression correlates with increased invasive potential [132]. Immunohistochemistry analysis revealed that elevated levels of BSP in bone lesions were more frequently associated with PCa metastases with respect to pure osteolytic metastases from breast cancer [116]. Although BSP was also detected in secondary lesions developed at visceral sites including liver and lung, its expression was significantly lower than in skeletal sites [133]. Whether this difference could be an important determinant for mixed lytic/sclerotic lesion is not resolved. BSP represents a good substrate for migration of cancer cells expressing *α*v*β*3 and *α*v*β*5 integrins. Integrin binding to BSP strongly stimulates adhesion to extracellular bone matrix and proliferation of cancer cells [134, 135].

Osteonectin (ON), also known as secreted protein, acidic, cysteine-rich (SPARC), is a calcium-dependent collagen binding protein particularly abundant in the bone. Normal prostate epithelial cells express low levels of ON, but its expression is increased in metastatic sites [136]. ON expression is associated with activation of *α*v*β*3 and *α*v*β*5 integrins, release of metalloproteases, and increased invasiveness [137]. One study demonstrated that the presence of ON in bone extract stimulated migration of PCa cells [138]. However the function of ON in bone metastasis is poorly understood and controversial, since growing body of evidence suggests that ON expression in bone environment may limit cancer growth [139]. It was proposed that the antimetastatic role of ON was due to its ability to mask or modify type I collagen, which seems to be fundamental in promoting the proliferation of bone-metastatic cancer cells [140]. Alternatively, an ON-enriched environment could negatively influence osteoclastogenesis, blocking the formation of the vicious cycle [139]. For this reason, after migration in the bone, the proliferation of PCa cells could be dependent on the enzymatic processing of ON. Indeed, it was shown that cathepsin K released by PCa cells is able to cleave ON [141].

OC, alias bone gamma-carboxyglutamic acid containing protein (BGLAP) is a noncollagenous, vitamin K-dependent protein secreted by osteoblasts in the late stage of their differentiation and is often used as a serum marker for increased bone mineral density. Its transcriptional expression is positively regulated by Runx2. About the specific functions in bone, OC regulates bone remodeling by modulating osteoblasts and osteoclast activity. Moreover, it also acts as a regulator of bone mineralization. OC can be found as a carboxylated or undercarboxylated form, the latter having an endocrine role, able to influence extraskeletal functions, such as energy metabolism, male fertility, and cognitive functions [142]. Although OC is expressed in the majority of PCa cells that metastasize the bone, its driver role in tumor progression is not yet clearly demonstrated, and it is currently studied for its role as serum marker of bone remodeling associated with PCa bone metastases.

### 7.6. Notch/Jagged Pathway

The potential involvement of this pathway in PCa bone metastases arises fromrecent evidence showing that metastatic PCa cells have increased levels of jagged-1 compared to benign prostatic tissues or to the primary tumor; on the contrary, it has been reported that the expression of its receptor notch-1 was significantly elevated in human PCa bone metastases [143]. Notch and hedgehog signaling have been recently found to be involved in the maintenance of PCa tumor-initiating cells [144].

### 7.7. Annexin II

Another potential marker recently discovered to be important for PCa homing to bone is annexin II. Starting from the evidence that annexin II, expressed by endothelial cells and osteoblasts, is important for their role in the formation of hematopoietic niche [145], it has been demonstrated that PCa cells migrate toward annexin II. In addition, annexin II inhibitors prevent PCa cells adhesion to osteoblasts and endothelial cells, thus strongly suggesting that PCa cells employ the same mechanisms of hematopoietic stem cells to gain access to the bone niche [146].

## 8. Conclusions

Because PCa is a slow growing cancer of the elderly, in order to decrease the associated mortality, the prevention of metastases is particularly important. Among the different metastatic diseases, bone lesions warrant the best benefit from current therapeutic choices. The recent approved therapies for CRPC, including new chemotherapy and hormonal deprivation agents, have shown significant improvements in overall survival and delayed time to bone metastasis. In parallel, our knowledge about the molecular factors determining high osteotropism is progressively increasing. Although there are important limitations in the data obtained from available preclinical models, the affinity of PCa cells for the bone has been consistently associated with the crosstalk between cancer and bone marrow cells. The better molecular definition of such interactions suggests a future in which the optimum combination of target therapy and chemotherapy will assure a decisive delay in the onset of clinically relevant bone metastasis.
